# Influenza A-Triggered Severe Acute Chest Syndrome in a Child With Sickle Cell Disease Complicated by Plastic Bronchitis and Posterior Reversible Encephalopathy Syndrome

**DOI:** 10.7759/cureus.105906

**Published:** 2026-03-26

**Authors:** Abdullah B Alshehri, Hassan Alamri, Sara S Hassanien, Ahlam A Babiker, Mohammed Alimamali, Mohammed Shakir, Ehab Hanafy

**Affiliations:** 1 Pediatrics Critical Care Unit, Armed Forces Hospital Southern Region, Khamis Mushayt, SAU; 2 Pediatric Pulmonology, Armed Forces Hospital Southern Region, Khamis Mushayt, SAU; 3 Oncology Center, Armed Forces Hospital Southern Region, Khamis Mushayt, SAU

**Keywords:** acute chest syndrome, influenza a, pediatric, plastic bronchitis, posterior reversible encephalopathy syndrome, saudi arabia, sickle cell disease

## Abstract

Acute chest syndrome (ACS) is one of the leading causes of morbidity and mortality in children with sickle cell disease (SCD) and is commonly precipitated by respiratory infections. Among viral triggers, influenza A is a recognized cause of severe ACS and may contribute to rapid clinical deterioration.

We report a case of a nine-year-old girl with homozygous sickle cell disease (HbSS), with irregular follow-up and poor adherence to hydroxyurea therapy, who presented with severe influenza A-associated ACS. Her course was complicated by plastic bronchitis, posterior reversible encephalopathy syndrome (PRES), and markedly elevated transcranial Doppler velocities. She required admission to the pediatric intensive care unit, bronchoscopy with removal of bronchial casts, transfusion therapy, and coordinated multidisciplinary management. The case illustrates a severe and unusual constellation of pulmonary and neurological complications occurring in the setting of poorly controlled SCD.

This case highlights the potential for influenza A to precipitate life-threatening ACS in children with SCD, particularly in the context of suboptimal disease control. It also underscores the complex interplay between pulmonary and neurological complications and emphasizes the importance of early recognition, timely escalation of care, and multidisciplinary management in severe presentations.

## Introduction

Sickle cell disease (SCD) is an inherited hemoglobinopathy characterized by chronic hemolysis, recurrent vaso-occlusive events, and progressive end-organ damage. Despite advances in preventive and disease-modifying therapies, children with SCD remain at risk for acute, potentially life-threatening complications. Acute chest syndrome (ACS) is the most common cause of hospitalization and one of the leading causes of death in pediatric SCD, often triggered by infections, particularly viral respiratory pathogens such as influenza A [1].

Influenza infection in children with SCD is associated with increased rates of hospitalization, severe ACS, intensive care admission, and mortality, even among vaccinated individuals. Viral-induced pulmonary inflammation promotes hypoxia, endothelial activation, and vaso-occlusion, thereby triggering a cascade that may precipitate severe systemic complications [2].

Hydroxyurea therapy significantly reduces the incidence of ACS and other SCD-related complications; however, poor adherence remains a major barrier to optimal disease control [3]. In rare circumstances, severe ACS may be complicated by unusual and life-threatening manifestations, including plastic bronchitis and neurological involvement.

Plastic bronchitis is characterized by the formation of firm, branching bronchial casts that obstruct the airways and has rarely been described in association with ACS in SCD [4]. Posterior reversible encephalopathy syndrome (PRES), a clinico-radiological entity characterized by vasogenic edema, is increasingly recognized in pediatric SCD, particularly in the setting of hypoxia, acute anemia, infection, or transfusion [5]. Elevated transcranial Doppler (TCD) velocities are a well-established marker of increased ischemic stroke risk in children with SCD [6].

The coexistence of influenza A-triggered ACS, plastic bronchitis, PRES, and elevated TCD velocities in a single pediatric patient is exceedingly rare and highlights the catastrophic potential of poorly controlled disease.

## Case presentation

A nine-year-old girl with known homozygous sickle cell disease (HbSS) presented with cough, shortness of breath, and chest pain. She was diagnosed with a vaso-occlusive crisis precipitated by influenza A infection. Initial management included supplemental oxygen via nasal cannula, nebulized bronchodilators, antiviral therapy with oseltamivir, intravenous hydration, and analgesia. Initial laboratory investigations demonstrated anemia, leukocytosis, thrombocytosis, and biochemical abnormalities, including hypophosphatemia, hypoalbuminemia, and elevated gamma-glutamyl transferase (GGT), as summarized in Table 1.

**Table 1:** Initial laboratory investigations on admission. WBC, white blood cell count; RBC, red blood cell count; Hb, hemoglobin; ANC, absolute neutrophil count; ALT, alanine aminotransferase; AST, aspartate aminotransferase; GGT, gamma-glutamyl transferase; BUN, blood urea nitrogen; Hb A, hemoglobin A; Hb A2, hemoglobin A2; Hb S, sickle hemoglobin; Hb F, fetal hemoglobin.

Parameter	Value	Reference/normal value
WBC	19.40 ×10^9/L	4.5-13.5 ×10^9/L
RBC	2.83 ×10^12/L	4.1-5.3 ×10^12/L
Hb	8.08 g/dL	10.9-15 g/dL
Platelets	694 ×10^9/L	140-450 ×10^9/L
ANC	12.04 ×10^9/L	1.5-8.5 ×10^9/L
Calcium, serum	2.19 mmol/L	2.22-2.57 mmol/L
Albumin	27.49 g/L	35-48 g/L
Magnesium, serum	0.64 mmol/L	0.66-1.07 mmol/L
Phosphorus, serum	0.22 mmol/L	0.94-1.91 mmol/L
Alkaline phosphatase	102 U/L	118-360 U/L
Corrected calcium	2.44 mmol/L	2.2-2.5 mmol/L
Total protein	56.00 g/L	65-83 g/L
Total bilirubin	15.60 µmol/L	<34.2 µmol/L
Albumin	27.49 g/L	31-48 g/L
Direct bilirubin	6.20 µmol/L	1.7-8.6 µmol/L
ALT	17 U/L	11-28 U/L
AST	34 U/L	21-36 U/L
Alkaline phosphatase	102.00 U/L	118-360 U/L
GGT	50.00 U/L	6-19 U/L
Sodium, serum	143.00 mmol/L	135-143 mmol/L
Potassium, serum	3.40 mmol/L	3.4-5.4 mmol/L
Chloride, serum	101.00 mmol/L	99-114 mmol/L
Creatinine, serum	39.10 µmol/L	34-97 µmol/L
BUN	3.50 mmol/L	2.5-6 mmol/L
Procalcitonin	0.30 ng/mL	<0.1 ng/mL
Hb A2	3.0%	1-3.5%
Hb A	24.7%	96.5-99%
Hb S	70%	0
Hb F	2.3%	2%

**Figure 1:**
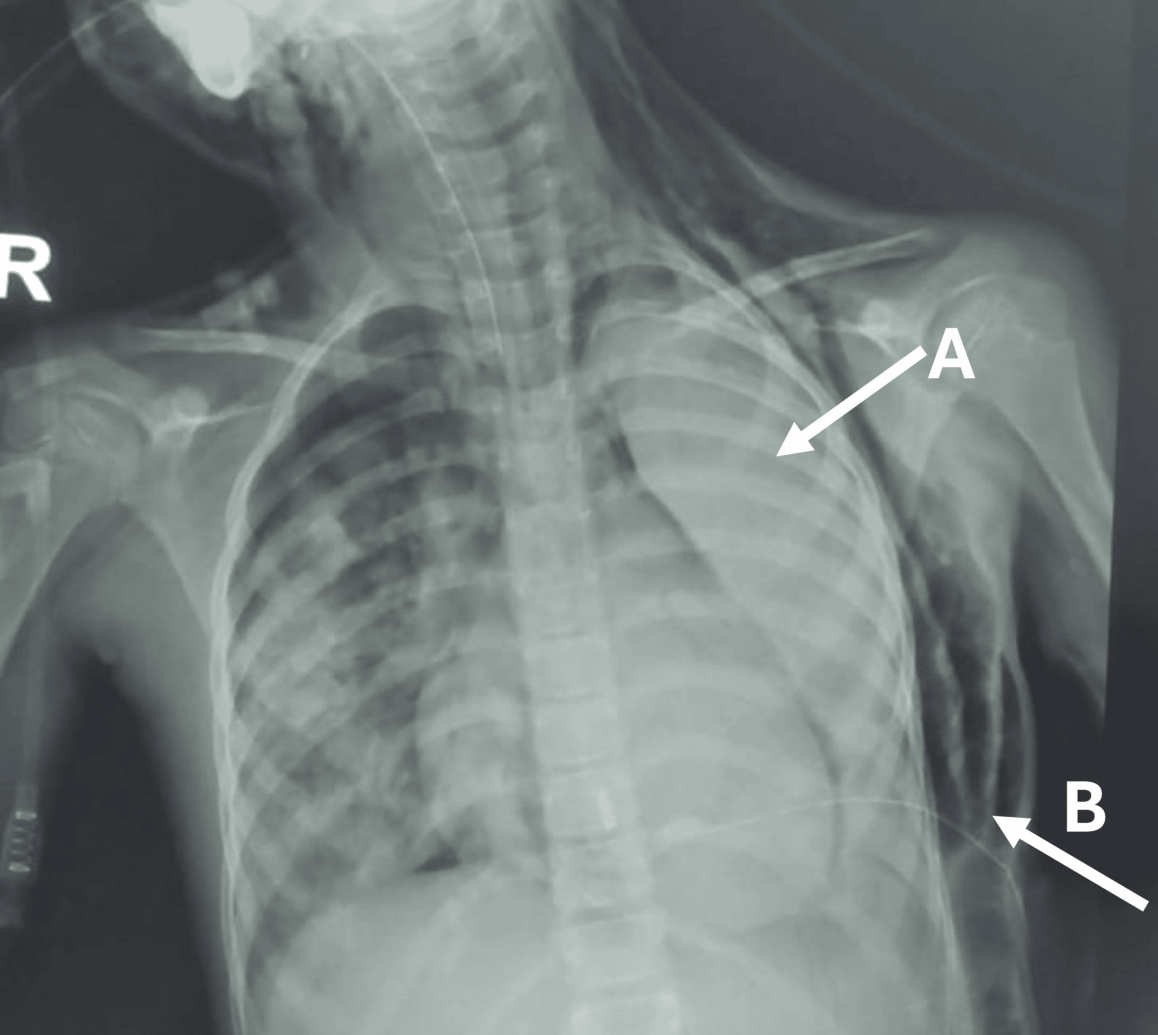
Chest radiograph during severe acute chest syndrome. Chest radiograph obtained during clinical deterioration showing near-complete opacification of the left hemithorax (arrow A) consistent with complete left lung collapse, with associated left-sided pleural pathology and subcutaneous emphysema of the left chest wall (arrow B) in the setting of severe acute chest syndrome.

On the fourth day of hospitalization, her clinical condition deteriorated with the development of ACS. Imaging demonstrated massive left-sided pleural effusion, minimal right-sided pleural effusion, complete collapse of the left lung, and progressive hypoxia with escalating oxygen requirements (Figure 1). Within 24 hours, she required endotracheal intubation and mechanical ventilation, consistent with a severe ACS phenotype characterized by progressive pulmonary involvement and worsening hypoxemia.

During her pediatric intensive care unit (PICU) course, multiple complications occurred. From a respiratory perspective, a left intercostal chest drain was inserted for pleural effusion drainage. Owing to refractory hypoxemia, ventilatory support was escalated to high-frequency oscillatory ventilation. Her course was further complicated by air leak syndrome and severe surgical emphysema, necessitating insertion of additional high intercostal chest drains.

From a cardiovascular standpoint, she developed premature ventricular contractions, which were managed with beta-blocker therapy titrated to optimal response. Her baseline hemoglobin S (HbS) level was 70%, and an exchange transfusion was performed, resulting in a post-exchange HbS level of 20%, in accordance with guideline-based management of severe ACS. Given the severity of her presentation and concern for severe sepsis, she also received broad-spectrum antibiotic therapy covering gram-positive, gram-negative, anaerobic, and atypical organisms, as well as early intravenous immunoglobulin (IVIG).

**Figure 2:**
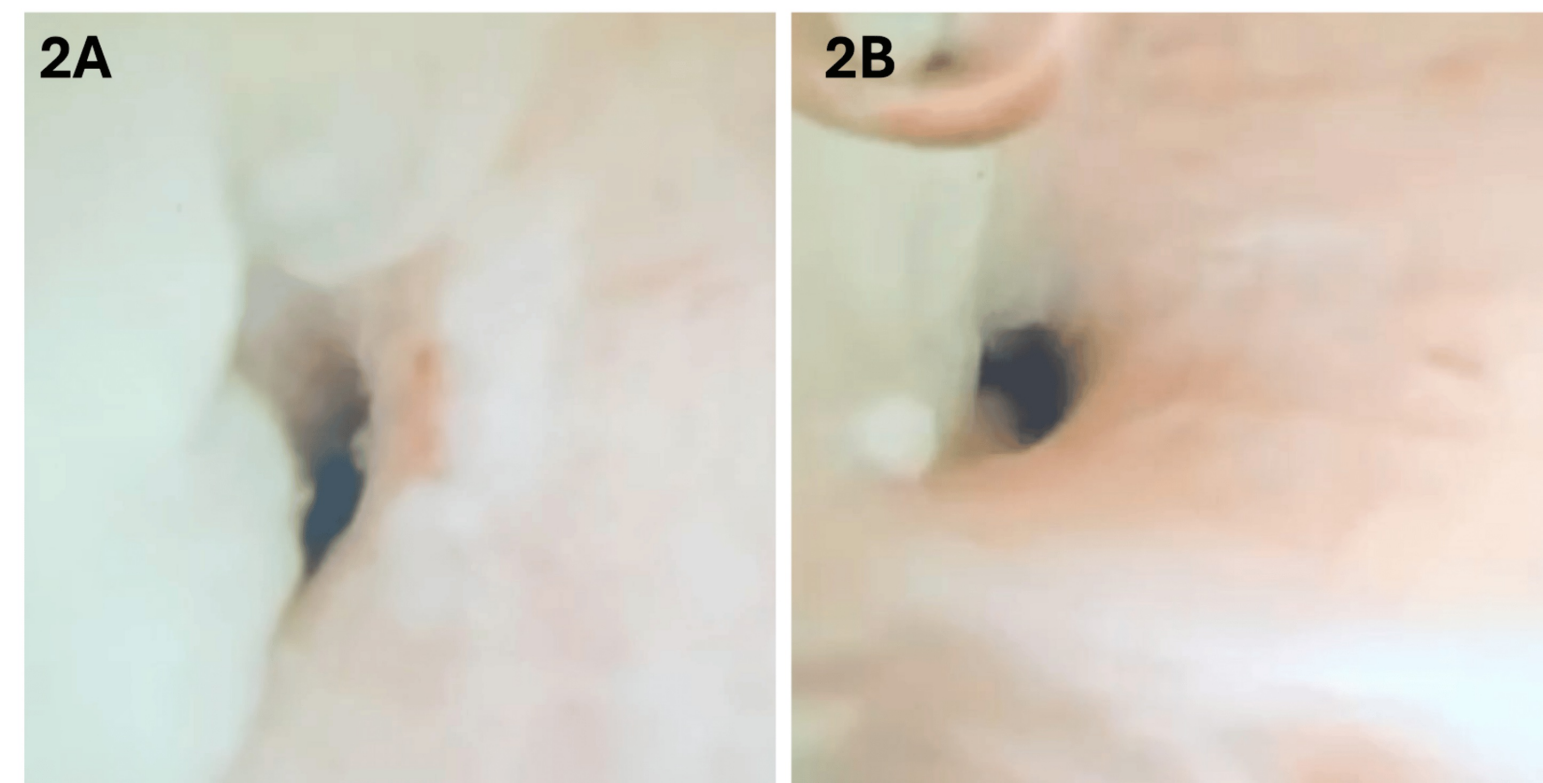
Bronchoscopic view of the endobronchial obstruction. Bronchoscopic images demonstrating obstructing endobronchial cast material within the left main bronchus, consistent with plastic bronchitis complicating severe acute chest syndrome. (A) Initial bronchoscopic view showing airway obstruction by thick cast material. (B) Bronchoscopic view during therapeutic removal of the obstructing cast.

The patient subsequently developed complete left lung collapse, prompting flexible bronchoscopy, which confirmed the presence of large, firm, rubbery, branching casts obstructing the left bronchial tree and causing complete airway blockage, consistent with plastic bronchitis (Figure 2).

**Figure 3:**
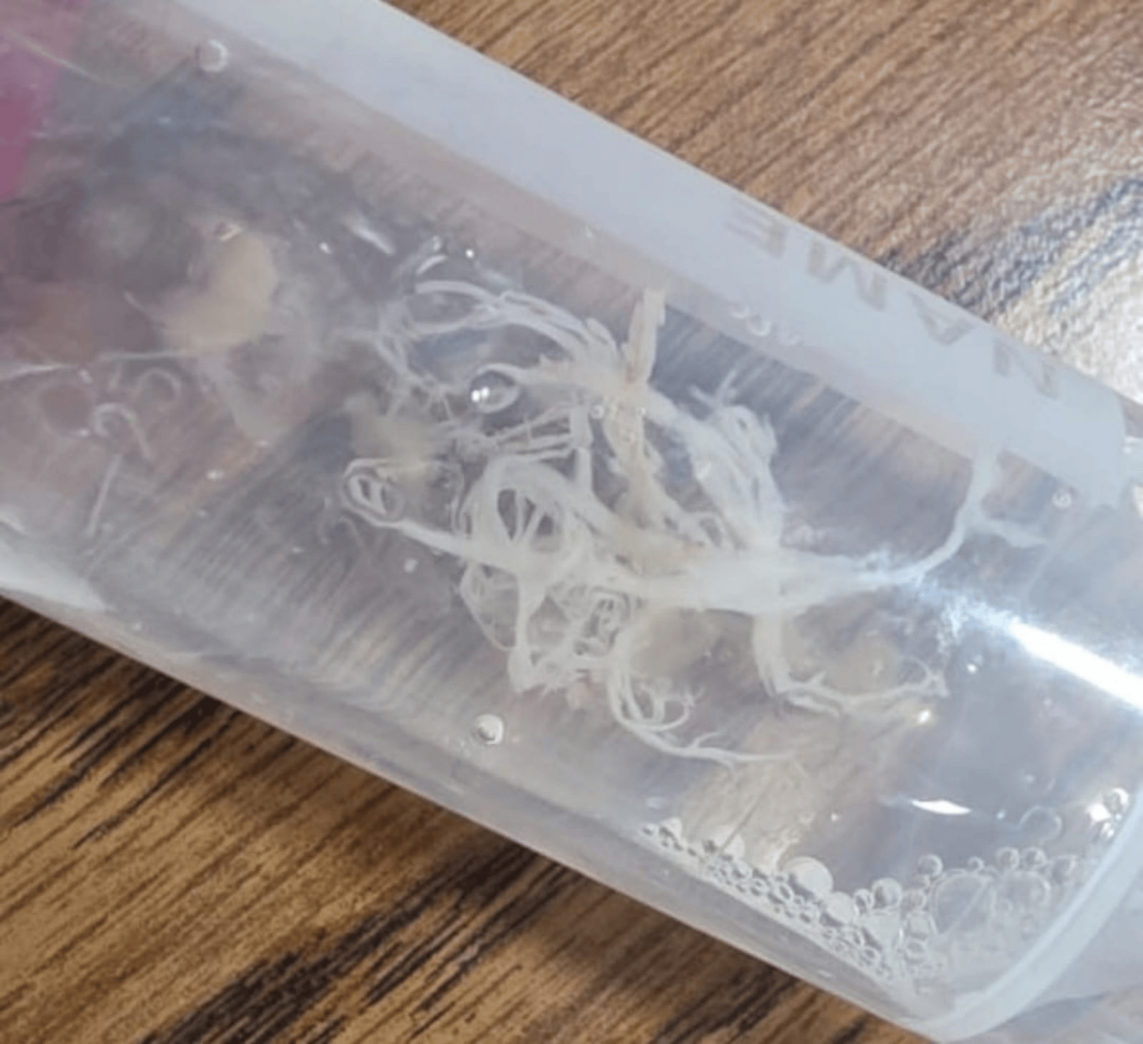
Extracted bronchial cast. Extracted branching fibrinous bronchial cast removed during therapeutic bronchoscopy, confirming plastic bronchitis.

Whole left lung lavage was performed in two sessions. During the first session, endobronchial instillation of alfa dornase 2.5 mg and N-acetylcysteine 3 mL was attempted; however, the procedure had to be interrupted because of severe hypoxia. Two days later, a second whole-lung lavage was performed with endobronchial instillation of alfa dornase 2.5 mg administered twice, N-acetylcysteine 3 mL administered twice, and normal saline 20 mL administered four times. The procedure lasted approximately 30 minutes. Therapeutic bronchoscopy with lavage and removal of obstructing fibrinous casts resulted in gradual improvement in ventilator requirements, and the left lung collapse progressively resolved over the following three days until extubation (Figure 3).

In this setting, plastic bronchitis represented a rare but serious complication of severe ACS, characterized by the formation of fibrinous endobronchial casts causing major airway obstruction, complete lung collapse, and prolonged ventilator dependence.

Following respiratory stabilization and gradual weaning of mechanical ventilation, she was noted to have significant generalized weakness, marked hypotonia, and intermittent hypertension. Neurological examination revealed bilateral lower motor neuron facial palsy and left-sided oculomotor nerve palsy, while the remaining cranial nerves were intact; these cranial nerve findings were described as fluctuating and atypical. She also had bilateral symmetrical quadriparesis, with motor power of approximately 1+/5 in all four limbs, marked generalized hypotonia, bilateral clawing of the hands and feet, generalized muscle wasting, and globally absent deep tendon reflexes.

**Figure 4:**
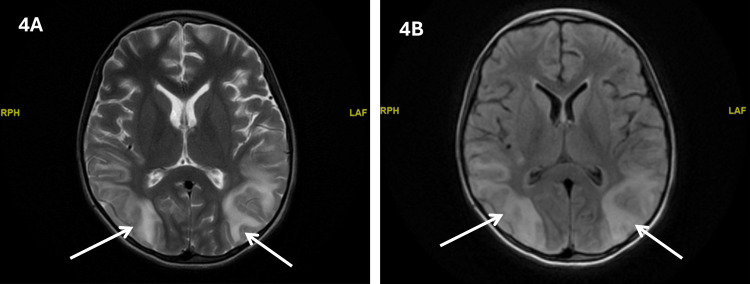
Brain MRI suggestive of posterior reversable encephalopathy. (A) Axial T2-weighted brain MRI showing bilateral posterior parieto-occipital cortico-subcortical hyperintense areas (white arrows), in keeping with vasogenic edema/posterior reversible encephalopathy (PRES) pattern. (B) Axial FLAIR (fluid-attenuated inversion recovery) image demonstrating symmetric posterior cortical-subcortical hyperintensity in the parieto-occipital regions (white arrows), compatible with PRES.

Magnetic resonance imaging (MRI) of the brain demonstrated high T2 signal intensity lesions involving the bilateral cortical and subcortical regions, most prominently in the occipital lobes, without diffusion restriction and without post-contrast enhancement (Figure 4). Magnetic resonance angiography and magnetic resonance venography were normal. The neuroradiological impression was PRES. Follow-up MRI confirmed PRES with interval evolution, showing faint foci of diffusion restriction in the parieto-occipital regions and scattered micro-hemorrhagic foci. The neurological picture was therefore considered consistent with PRES in association with critical illness neuropathy.

She was managed with blood pressure control, optimization of fluid balance, and careful oxygenation and ventilation strategies. Her PICU course was further complicated by prolonged mechanical ventilation requiring gradual weaning, as well as nutritional challenges necessitating optimization of enteral feeding. She also required intensive physiotherapy. Over time, her respiratory status improved, with resolution of lung infiltrates, successful extubation, and progressive neurological recovery, and her total PICU length of stay was 34 days.

She was subsequently transferred from the PICU to the general pediatric ward in stable condition on room air. At discharge, she had no residual respiratory compromise, her blood pressure was well controlled without the need for long-term antihypertensive therapy, and her neurological status had improved significantly with physiotherapy. Repeat brain MRI at follow-up demonstrated near-complete resolution of the prior PRES-related changes.

## Discussion

This case illustrates the devastating interplay between viral infection, poor baseline disease control, and systemic inflammation in pediatric SCD. Influenza A appears to have acted as the principal trigger for severe ACS, initiating a cascade of pulmonary deterioration followed by major neurological complications. Respiratory viral infections are among the most common precipitants of ACS, and influenza has been associated with particularly severe disease in children with SCD, including higher rates of hospitalization, intensive care admission, respiratory failure, and death compared with the general pediatric population [1,2]. In the present patient, influenza A likely amplified pulmonary inflammation, endothelial injury, ventilation-perfusion mismatch, and hypoxia, thereby accelerating progression from vaso-occlusive crisis to severe ACS.

The respiratory course was further complicated by plastic bronchitis, a rare but potentially fatal manifestation of ACS in SCD. Plastic bronchitis is characterized by the formation of large branching endobronchial casts that obstruct major airways and may result in persistent atelectasis, severe hypoxemia, and prolonged ventilatory dependence [4]. Similar presentations have been described in the literature, including adult and pediatric cases in which bronchoscopy was required to establish the diagnosis after failure of conventional ACS treatment [7,8]. More recent reports have further emphasized the severity of this complication, including pediatric cases with catastrophic outcomes and reports describing rescue therapies such as inhaled tissue plasminogen activator in refractory disease [9]. In our patient, persistent left lung collapse despite conventional management raised concern for ongoing proximal airway obstruction, and flexible bronchoscopy ultimately demonstrated mucus plugging and fibrinous casts consistent with plastic bronchitis. The subsequent improvement after bronchial lavage and cast removal strongly supports the pathogenic role of endobronchial cast obstruction in her deterioration and highlights the importance of early bronchoscopic evaluation in cases of refractory hypoxemia or unresolved lung collapse.

The neurological complication in this case was equally significant. PRES is increasingly recognized in patients with SCD, although it remains uncommon and likely underdiagnosed outside settings of overt seizures or acute neurological decline [5]. Available reports suggest that PRES may occur in the setting of acute systemic decompensation and is probably multifactorial, involving endothelial dysfunction, hypoxia, acute anemia, infection, hypertension, and transfusion-related hemodynamic stress [10-12]. Additional evidence also suggests that PRES may be more frequent in hemoglobinopathy settings characterized by major vascular stress, further supporting the concept that cerebral autoregulatory failure in SCD is often not attributable to a single trigger alone [11]. In the present case, intermittent hypertension was documented, but the clinical context suggests a broader multifactorial insult rather than isolated blood pressure elevation alone. Severe influenza-associated ACS likely produced profound hypoxic and inflammatory stress, while critical illness and transfusion may have further contributed to endothelial injury and cerebral dysregulation [5,11,12].

The MRI pattern in this patient was highly compatible with PRES, showing bilateral cortical and subcortical T2 hyperintensities, predominantly in the occipital regions, with subsequent interval evolution and later near-complete radiological resolution. This reversibility, together with improvement after supportive management, blood pressure control, and stabilization of the underlying systemic illness, further supports the diagnosis. Importantly, timely neuroimaging in such patients is essential because neurological manifestations in SCD can easily be misattributed solely to stroke, metabolic derangement, sedation-related effects, or critical illness neuropathy [5,11].

The markedly elevated TCD velocities observed in this patient add another important dimension to the case. Elevated TCD velocities are a well-established marker of increased ischemic stroke risk in children with SCD, and abnormal screening results identify patients who benefit from stroke-prevention strategies [6]. Although TCD findings may be influenced by acute physiological stress, the markedly abnormal velocities in this setting likely reflect substantial cerebrovascular vulnerability during severe systemic illness. Large clinical trials have shown that chronic transfusion therapy reduces stroke risk in children with abnormal TCD findings, while hydroxyurea can maintain TCD velocities in selected patients after an initial period of transfusion therapy when severe vasculopathy is absent [13]. In this context, the TCD findings reinforce the importance of structured follow-up, standardized cerebrovascular screening, and aggressive secondary prevention planning after recovery from the acute event.

Another central lesson from this case is the role of poor baseline disease control. Hydroxyurea is one of the most effective disease-modifying therapies in SCD and has been consistently associated with a reduction in ACS, vaso-occlusive events, and other serious complications [3]. In our patient, irregular follow-up and poor adherence to hydroxyurea likely increased baseline vulnerability to severe inflammatory and vaso-occlusive complications once influenza infection occurred. The resulting presentation was not merely severe ACS, but rather a convergence of severe pulmonary disease, airway cast formation, prolonged respiratory failure, PRES, and cerebrovascular risk markers.

The simultaneous occurrence of severe influenza A-triggered ACS, plastic bronchitis, PRES, and markedly elevated TCD velocities in a single child with SCD is exceptionally uncommon and illustrates the extent to which acute infection can precipitate multisystem decompensation in an already vulnerable patient. This case underscores the need for heightened clinical suspicion when children with SCD deteriorate despite standard therapy, particularly when respiratory failure is refractory or new neurological findings emerge. Early recognition of unusual complications, together with timely multidisciplinary intervention, is essential to reduce the risk of irreversible morbidity and improve clinical outcomes.

## Conclusions

Influenza A infection can precipitate severe ACS with rapid progression to life-threatening multisystem complications in children with SCD. In this case, poor baseline disease control and irregular hydroxyurea adherence likely increased vulnerability to an unusually severe course characterized by respiratory failure, plastic bronchitis, PRES, and abnormal cerebrovascular findings. The case highlights how pulmonary and neurological complications may coexist and evolve during the same acute illness.

This report emphasizes the importance of prevention and early escalation of care in high-risk pediatric SCD. Vaccination, adherence to disease-modifying therapy, prompt antiviral treatment, close respiratory and neurological monitoring, and early multidisciplinary management are critical to improving outcomes. Clinicians should also maintain a low threshold for bronchoscopy in refractory lung collapse and for neuroimaging when unexplained neurological abnormalities develop during severe ACS.
